# Socioeconomic vulnerability associated with toxocariasis exposure in southern Brazil: a One Health approach

**DOI:** 10.3389/fpubh.2026.1759431

**Published:** 2026-03-18

**Authors:** Natacha Sohn-Hausner, Ricardo Guedes Correa, Susana Angélica Zevallos Lescano, Paula Sabrina Brime, Rogério Giuffrida, Louise Bach Kmetiuk, Vamilton Alvares Santarém, Alexander Welker Biondo

**Affiliations:** 1 Graduate College of Cell and Molecular Biology, Federal University of Paraná, Curitiba, PR, Brazil; 2 Department of Planning, Conservation and Environmental Education, Municipal Secretary of the Environment, Pinhais, Paraná, Brazil; 3 Graduate College of Veterinary Medicine, Federal University of Paraná, Curitiba, PR, Brazil; 4 Institute of Tropical Medicine, University of São Paulo, São Paulo, Brazil; 5 Graduate College in Health Sciences and in Animal Sciences, University of Western São Paulo (UNOESTE), São Paulo, Brazil; 6 Center of Environmental Health, Municipal Secretary of Health, Curitiba, Paraná, Brazil

**Keywords:** neglected tropical diseases, neglected zoonosis, One Health, social determinants of health, socioeconomic vulnerability

## Abstract

**Introduction:**

Although toxocariasis has been listed among the five most neglected parasitic infections worldwide, with higher impact in socioeconomically vulnerable populations, no study has evaluated vulnerable individuals from highly populated urban areas.

**Methods:**

Accordingly, human serum samples were collected in Curitiba, the eighth most populated metropolitan area of Brazil, and tested by indirect enzyme-linked immunosorbent assay (ELISA) for anti-*Toxocara* spp. antibodies. Sociodemographic and environmental information was collected by epidemiological questionnaires and geospatial data to assess associated risk factors and toxocariasis areas.

**Results:**

Overall, 58 of 132 (43.9%) (95% CI: 35.8–52.5%) human samples were seropositive for *Toxocara* spp., with the final multivariate logistic regression model identifying age as the only predictive factor associated with seropositivity. Individuals aged 53 to 80 years had a 3.46 times higher risk (OR = 3.46; 95% CI: 1.24–10.17; *p* < 0.05) when compared to the 3 to 20 years group. Kernel analysis revealed an area with a higher density of seropositive individuals in locations lacking access to basic sanitation, highlighting a spatial risk area for *Toxocara* spp. transmission.

**Discussion:**

Although age over 53 years was the only predictor in the final logistic regression model, the spatial overlap between asymptomatic seropositive individuals and areas of high environmental vulnerability—marked by low income, flooding, and poor sanitation—was critical. These conditions likely facilitated the *Toxocara* spp. life cycle, making the identification of such areas pivotal for local public health, effectively targeting toxocariasis intervention and control strategies. In addition, the One Health approach herein highlights human vulnerability to toxocariasis as a life-course risk, beyond pet healthcare and environmental exposure in endemic areas.

## 1 Introduction

Neglected tropical diseases affect approximately two billion people worldwide and annually cause approximately 177,000 deaths (1, 2). Although responsible for a high morbidity and mortality burden, particularly in developing countries, these diseases have historically received insufficient investment in research and control efforts (3). According to the US Centers for Disease Control and Prevention (CDC), toxocariasis, caused by the helminths *Toxocara canis* and *Toxocara cati*, has been listed among the five most neglected parasitic infections in the world. Infection normally occurs after accidental ingestion of embryonated eggs, releasing larvae that migrate through various internal tissues (4, 5). Clinical manifestations include eosinophilia, asthma or wheezing, atopy, hepatomegaly, urticaria, stunted growth, ocular larva migrans, and visceral involvement (6). Nonetheless, seroconverted individuals may remain asymptomatic over time (4).

As previously shown in a literature review, Toxocara spp. seroprevalence rate was 19.0% (95%CI, 16.6–21.4%; 62,927/265,327), with the highest reported in Africa (37.7%; 25.7–50.6%) and the lowest in the Eastern Mediterranean (8.2%; 5.1–12.0%) (7). Lower income levels and Human Development Index (HDI) scores have been significantly associated with higher *Toxocara* spp. seroprevalence. In addition, male sex, living in a rural area, soil contact, childhood age, contact with cats and dogs, onychophagia, raw meat intake, and drinking untreated water have been frequently associated with *Toxocara* spp. seropositivity (7).

Higher *Toxocara* spp. seropositivity has been reportedly associated with socioeconomically vulnerable populations (8, 9). In Brazil, the USA, Venezuela, and Pakistan, low educational levels and income were frequently associated with *Toxocara* spp. exposure, especially when combined with environmentally associated risk factors. In Brazil, seroprevalence among children and school-aged populations living in urban, semi-rural, and rural settings varied from 9.7% to 63.6% (4). Key risk factors included contact with dogs, cats, or puppies; soil exposure (notably in recreational areas); abnormal behaviors such as geophagy or onychophagia; and socioeconomic indicators such as poor hygiene and low maternal education (5, 6).

Such critical synthesis of existing studies has become very important, particularly with regard to the marked heterogeneity in seroprevalence across different settings. Urban vulnerability remains an important knowledge gap, especially in metropolitan areas of major cities in developing countries. The One Health perspective has been proposed to be fully integrated into the analytical framework, as the convergence of human, animal, and environmental dimensions may provide new insights for understanding the disease (10). Our research group has shown the advantages of such an approach to toxocariasis in other vulnerable populations, such as indigenous (8, 11), *quilombola*—former slaves (12), incarcerated (13), homeless (14), traditional seashore fishermen (15), as well as schoolchildren (16), elderly (17), pregnant women (18), and animal-hoarding individuals (19). In this context, the One Health approach to toxocariasis in vulnerable urban settings should extend beyond considering toxocariasis as only a pet healthcare issue or an environmental exposure problem, particularly in endemic areas.

Anti-*Toxocara* spp. antibodies in elderly individuals may be a consequence of the continuing and cumulative effects of *Toxocara* exposure during their lifetimes (17). In other words, toxocariasis risk would be translated in terms of cumulative or age-related exposure trajectories. However, age-related exposure is still controversial, as no statistical differences were observed when comparing age groups, likely an age-independent factor, in recent studies of Iranian (20, 21) and Brazilian elderly (17). Meanwhile, a survey in *quilombola* (former slave) communities has shown that individuals over 50 years of age were 7.7-fold more likely to be seropositive, probably due to maintenance of anti-*Toxocara* spp. antibody levels caused by exposure over time to infection via soil in daily agricultural activities (12). Thus, this pattern in vulnerable populations living in densely populated urban areas of developing countries remains to be fully established.

Although previous studies have suggested that higher *Toxocara* spp. seroprevalence has been associated with lower income, no study to date has evaluated whether socioeconomic determinants interact with environmental factors, such as untreated river water, inadequate sanitation, overcrowded living spaces, and animal contact, to increase toxocariasis risk in urban areas. Accordingly, the present study aimed to assess *Toxocara* spp. seropositivity in socioeconomically vulnerable individuals in Pinhais, a municipality in Curitiba, the eighth most populated metropolitan region of southern Brazil, from a One Health perspective.

## 2 Methods

### 2.1 Ethical statement

This study was approved by the National Research Ethics Commission of the Brazilian Ministry of Health (Protocol No. 34934220.4.0000.0102) and by the Animal Use Ethics Committee of the Federal University of Paraná (Protocol No. 078).

### 2.2 Study area

This study was conducted in the eighth most populous metropolitan region of Brazil, in the municipality of Pinhais (25°25′57^′′^ S, 49°11′35^′′^ W), located in the state of Paraná, southern Brazil. Pinhais is part of the Curitiba metropolitan area, which comprises approximately 3.73 million inhabitants (22) and is ranked as the second-largest metropolitan area in Brazil by territorial extension, covering 16,581.21 km^2^ (23). According to the Köppen climate classification, the region has a subtropical highland climate (Cfb) climate, characterized by an annual mean temperature of 17 °C and an average annual precipitation of 1,550 mm (24). At the time, Pinhais was administratively divided into 15 neighborhoods at the time and encompassed four distinct hydrographic regions—corresponding to the Iraí, do Meio, Palmital, and Atuba river basins—each exhibiting varied environmental and demographic patterns (25).

### 2.3 Sample selection and study population

The study population was identified through a retrospective analysis of public records from the Municipal Health Department of Pinhais, spanning from 2007 to 2020. Initially, 5,973 official protocols related to household tick infestation complaints were identified. After screening for unique locations, 200 distinct addresses were selected for further eligibility assessment.

During the recruitment phase, 146 residents were successfully reached by telephone and initially agreed to participate in the study. However, due to the COVID-19 pandemic safety protocols and local health guidelines, predefined exclusion criteria were strictly applied during the field collection phase. A total of 74 addresses were excluded for the following reasons:

- Presence of residents with high-risk comorbidities for COVID-19;
- Withdrawal of consent or refusal of household entry on the collection day;
- Resident relocation (change of address);
- Absence of residents at the scheduled time.

The final analytical sample consisted of 135 human blood samples collected from 72 distinct households (Supplementary Figure 1). All collection procedures followed standard biosafety practices to ensure the safety of both researchers and the community during the pandemic period.

### 2.4 Blood sampling

A convenience-sampling approach was used, based on household tick infestation reports filed with the Pinhais Municipal Health Department. Sample collection was carried out by a multidisciplinary team between April 2019 and November 2020. All participants provided informed consent after receiving detailed explanations of the study objectives and procedures. Blood samples were obtained via venipuncture of the median cubital vein by licensed nurses, using collection tubes containing either ethylenediaminetetraacetic acid (EDTA) or separation gel. Following collection, the samples were centrifuged at 5,000 rpm for 5 min; the resulting serum was separated and stored at −20 °C until serological testing.

### 2.5 Pre-adsorption of sera with *Ascaris suum* adult worm extract and enzyme-linked immunosorbent assay (indirect ELISA)

Polystyrene 96-well microplates (Corning, Costar, Ref. 3590, New York) were coated with 1.9 μg/μL per well of *Toxocara* excretion–secretion (TES) antigens, derived from L3 larvae cultured in Roswell Park Memorial Institute (RPMI) medium. The coating was performed in 0.06 M carbonate-bicarbonate buffer (pH 9.6) and incubated for 2 h at 37 °C, followed by 18 h at 4 °C. The plates were then washed three times with phosphate-buffered solution (PBS) containing Tween 20 and blocked for 1 h at 37 °C with PBS-Tween supplemented with 5% skimmed milk (Molico^®^, Nestle, Vevey, Suíça).

Serum samples were pre-adsorbed with *Ascaris suum* somatic antigen (25 μg/mL) diluted in PBS-Tween with skimmed milk to minimize cross-reactivity (26). Subsequently, the samples were diluted 1:200 in the same solution, incubated at 37 °C for 30 min, and added in duplicate to the antigen-coated plates, followed by incubation at 37 °C for 1 h. After washing three times, a goat anti-human immunoglobulin G (IgG) peroxidase-conjugated antibody (Sigma A6029, Saint Louis, USA) was applied at a 1:5,000 dilution and incubated for 60 min at 37 °C. The plates underwent three washes of 5 min each.

The enzymatic reaction was developed using 3,3′,5,5′-tetramethylbenzidine (TMB) substrate (Thermo Fisher Scientific, Waltham, MA, USA) and stopped using 2 N sulfuric acid. Each plate included positive and negative control sera obtained from the serum bank of the Institute of Tropical Medicine, University of São Paulo, a diagnostic reference laboratory of tropical infectious diseases. Absorbance was measured at 450 nm, and the cutoff value was determined as the mean absorbance of 90 negative controls plus three standard deviations. The antibody levels were expressed as reactivity indices that were calculated as the ratio between the absorbance values of each test sample and the cutoff value. A serum sample was considered positive when its reactivity index was greater than 1 (27).

### 2.6 Epidemiological data collection

A standardized epidemiological questionnaire, developed based on previous studies and relevant literature, was administered to the volunteers to evaluate potential risk factors associated with *Toxocara* spp. exposure. The instrument consisted of closed-ended questions addressing variables associated with possible pathogen exposure, including demographic factors, such as gender and age; the presence of dogs and/or cats in the household; personal hygiene practices; and dietary habits.

Data regarding the population of Pinhais, organized by neighborhoods—including the number of elderly people receiving government assistance and per capita family income—were obtained at the Social Assistance Secretary of Pinhais. These data were derived from the Social Protection Coverage system, an auxiliary tool used for territorialization, monitoring, and registration of beneficiaries under the Unified National Social Assistance System (SUA) in Pinhais. The dataset was completed with the national information from the 2010 Demographic Census Brazilian Institute of Geography and Statistics (IBGE) and the 2020 Single Registry Unified Registry Information Consultation, Selection, and Extraction Tool (CECAD). These data were publicly available on the municipal government's website (28) and used in association analyses.

Geospatial data, including shapefiles and other geographic information such as leisure and sports areas, flood history, hydrography, sewage networks, forested areas, Permanent Preservation Areas (APP), neighborhood boundaries, municipal borders, and socioeconomic indicators, were obtained at the Urban Planning Department of Pinhais (29). These datasets were used to construct risk maps.

Individual-level variables collected from the epidemiological questionnaire were used for inferential statistical analyses. In contrast, neighborhood-level socioeconomic and environmental indicators were used exclusively for spatial and descriptive analyses to contextualize serological findings and identify geographic patterns, without individual-level inference.

All variables collected and analyzed in the study were systematically categorized according to their level of analysis (individual or neighborhood level), operational definition, and measurement scale (Supplementary Table 1).

### 2.7 Statistical analysis

Inferential statistical analyses were conducted exclusively at the individual level using variables obtained from the epidemiological questionnaire. Associations between potential risk factors and *Toxocara* spp. seropositivity were assessed using univariate analysis with the chi-square test or Fisher's exact test, as appropriate. Predictor variables with a *p*-value of < 0.20 in the univariate analysis were included in the multivariate logistic regression analysis. Odds ratios (OR) with 95% CI were calculated, and a *p*-value of < 0.05 was considered statistically significant. All analyses were conducted using R v. 4.2.2 (University of Auckland, New Zealand)(30).

Spatial analyses were performed for exploratory and descriptive purposes using the georeferenced locations of the residences where blood was collected. Thematic maps and cluster analyses, based on kernel density estimation, were generated using QGIS software tools (31). For kernel density, a 100-m radius was used to generate hotspot areas. To prevent the identification of individual residences, residential points were enlarged to cover the size of a city block rather than the size of individual residences. Street names or overlay points on the road network were not included to prevent the identification of the residence. Neighborhood boundaries, census areas, and spatial distribution of water and sewage networks were included to allow only the identification of the general region within a neighborhood, without revealing the location of specific sampled residences.

## 3 Results

### 3.1 Serological analysis

A total of 58 of the 132 individuals (43.90%, 95% CI: 35.76–52.46) were seropositive for *Toxocara* spp. (Tables 1, 2).

**Table 1:** Prevalence of IgG anti-*Toxocara* spp. antibodies in individuals from the municipality of Pinhais, southern Brazil.

**Variable**	**Results**	***N***	**%**	**CI 95%**	
				**Lower**	**Upper**
Seropositivity	Seropositive	58	43.90	35.76	52.46
	Seronegative	74	56.10	47.59	64.53

**Table 2:** Risk factors associated with a seropositive ELISA test for anti-*Toxocara* spp. evaluated by univariate and multivariate (logistic regression) analyses in a disadvantaged population of a large metropolitan city in southern Brazil.

**Variables**	**Serology**		**Univariate analysis**		**Multivariate analysis**	
	**Positive (%)**	**Negative (%)**	**OR (95% CI)**	***p*** **overall**	**OR (95% CI)**	***p***
Characteristics	58 (43.9)	74 (56.1)				
**Sex**				0.136		
Female	44 (75.9)	46 (62.2)	Ref.			
Male	14 (24.1)	28 (37.8)	0.53 (0.24–1.12)		0.58 (0.26–1.27)	0.179
**Age (years old)**				0.081		
3 up to 20	10 (17.2)	24 (32.4)	Ref.			
21 up to 36	15 (25.9)	19 (25.7)	1.87 (0.69–5.27)		1.74 (0.64–4.90)	0.285
37 up to 52	14 (24.1)	19 (25.7)	1.75 (0.63–4.97)		1.58 (0.57–4.50)	0.385
53 up to 80	19 (32.8)	12 (16.2)	3.69 (1.33–10.9)		3.46 (1.24–10.17)	0.020
**Dog ownership**				0.465		
No	2 (3.45)	5 (6.76)	Ref.			
Yes	56 (96.6)	69 (93.2)	1.94 (0.38–15.5)			
**Cat ownership**				0.9		
No	36 (62.1)	44 (59.5)	Ref.			
Yes	22 (37.9)	30 (40.5)	0.90 (0.44–1.82)			
**Ownership of both dogs and cats**				0.9		
No	36 (62.1)	44 (59.5)	Ref.			
Yes	22 (37.9)	30 (40.5)	0.90 (0.44–1.82)			
**Dog living status**				0.579		
No dog(s)	2 (3.45)	5 (6.76)	Ref.			
Stray dogs	5 (8.62)	9 (12.2)	1.34 (0.18–13.4)			
Domiciled dogs	51 (87.9)	60 (81.1)	2.03 (0.40–16.3)			
**Washing vegetables before meal**				0.919		
Water	35 (60.3)	44 (59.5)	Ref.			
Sodium hypochlorite	5 (8.62)	7 (9.46)	0.91 (0.24–3.16)			
Detergent	4 (6.90)	3 (4.05)	1.65 (0.32–9.45)			
Vinegar	14 (24.1)	20 (27.0)	0.88 (0.38–2.00)			
**Washing hands before meals**				0.244		
No	3 (5.36)	5 (6.76)	Ref.			
Water	13 (23.2)	21 (28.4)	1.02 (0.20–6.02)			
Water and soap	35 (62.5)	47 (63.5)	1.22 (0.27–6.66)			
Soap and alcohol	5 (8.93)	1 (1.35)	6.62 (0.61–234)			
**Raw meat consumption**				0.38		
No	47 (81.0)	54 (73.0)	Ref.			
Yes	11 (19.0)	20 (27.0)	0.64 (0.27–1.46)			
**Contact with soil**				0.226		
No	42 (72.4)	45 (60.8)	Ref.			
Yes	16 (27.6)	29 (39.2)	0.60 (0.28–1.24)			

### 3.2 Statistical analysis

The final logistic regression model revealed that age was the only predictive factor for human toxocariasis, with a 3.46-fold increased risk (95% CI: 1.24–10.17) of seropositivity for individuals aged 53–80 years compared to the age group of 3 to 20 years. Although the univariate analysis included gender in the logistic regression model (*p* = 0.136), this variable was not statistically significant in the final model (*p* = 0.179). Other risk factors were not statistically significant in the univariate analysis, including dog ownership (*p* = 0.465), cat ownership (*p* = 0.9), ownership of both dogs and cats (*p* = 0.09), dog living characteristics (*p* = 0.579), washing vegetables before meals (*p* = 0.919), washing hands before meals (*p* = 0.244), ingestion of raw meat (*p* = 0.38) and contact with soil (*p* = 0.226). Two missing data points were noted for the variable “washing hands” due to incomplete questionnaire responses.

**Figure 1:**
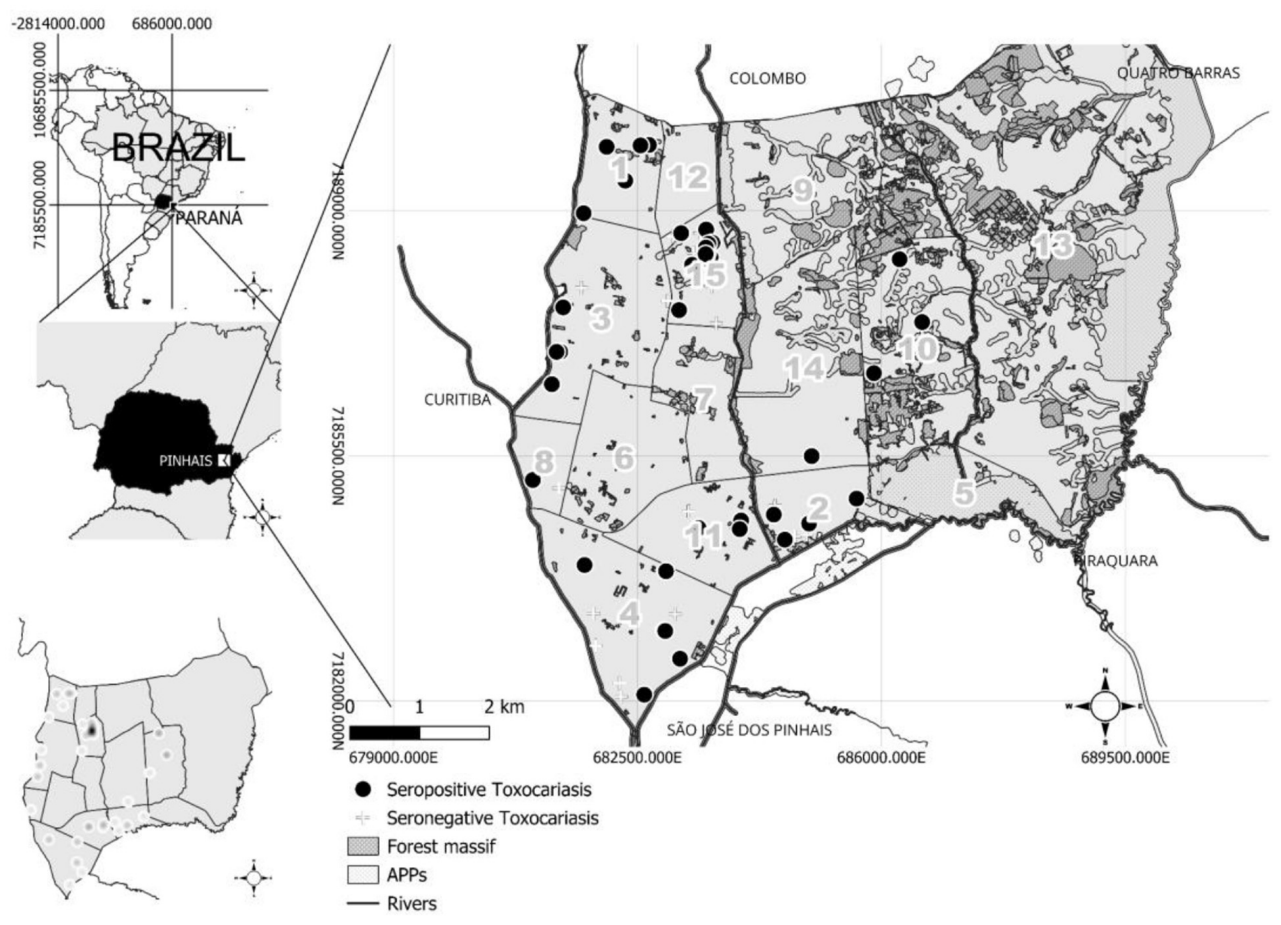
Location of seropositive and seronegative individuals for *Toxocara* spp., spatial density of seropositive individuals (kernel maps on the lower left) to *Toxocara* spp. in the municipality of Pinhais, southern Brazil. Neighborhoods: 1. Atuba, 2. Maria Antonieta, 3. Emiliano Perneta, 4. Weissópolis, 5. Parque das Águas, 6. Centro, 7. Pineville, 8. Estância Pinhais, 9. Alphaville Graciosa, 10. Jardim Karla, 11. Vargem Grande, 12. Jardim Cláudia, 13. Parque das Nascentes, 14. Jardim Amélia, and 15. Alto Tarumã.

Different denominators were used in the spatial analyses to reflect the number of individuals with valid geospatial linkage for each specific environmental or socioeconomic indicator. The serology results overlapped with historical flood levels, forest cover, and sewage and water networks to identify relationships and spatial patterns that are not visible through univariate and multivariate (logistic regression) analyses (Figure 1). A predominance of seropositive individuals was observed in the Alto Tarumã neighborhood (18/58; 31%), followed by Weissopolis (8/58; 13.8%), Atuba (7/58; 12.1%), Jardim Karla (7/58; 12.1%), Vargem Grande (6/58; 10.3%), Emiliano Perneta (5/58; 8.6%), Maria Antonieta (5/58; 8.6%), Jardim Amelia (1/58; 1.7%), and Estância Pinhais (1/58; 1.7%).

The kernel analysis was used to verify clustering patterns and revealed Alto Tarumã as a neighborhood with a higher density of seropositive individuals and a possible risk area for *Toxocara* spp. transmission. Six neighborhoods had no seropositive individuals (Figure 1).

**Figure 2:**
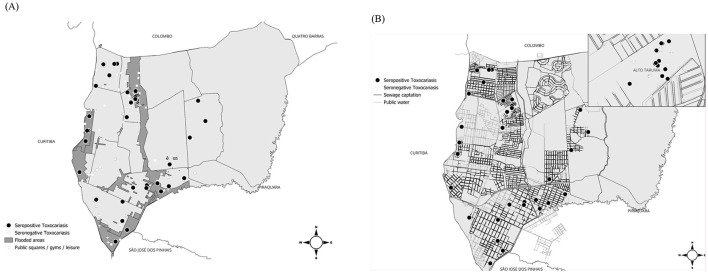
Areas with environmental risk for *Toxocara* spp. and serological results in Pinhais, Paraná, Brazil. In **(A)**, flood areas, public squares, gyms, and/or areas used for leisure and in **(B)**, water and sewage supply networks.

Areas considered at risk for environmental contamination by *Toxocara* spp. eggs, including parks, woods, sand courts, multi-sport courts, historical flooding areas, public sewage networks, and water supply networks spatially overlapped with serological results, included 39 of 99 (39.39%) individuals living in flooded areas, 58 of 132 (43.94%) living in low-income neighborhoods, and 37 of 95 (38.95%) living in neighborhoods with a higher number of elderly people receiving government assistance (Figure 2).

**Figure 3:**
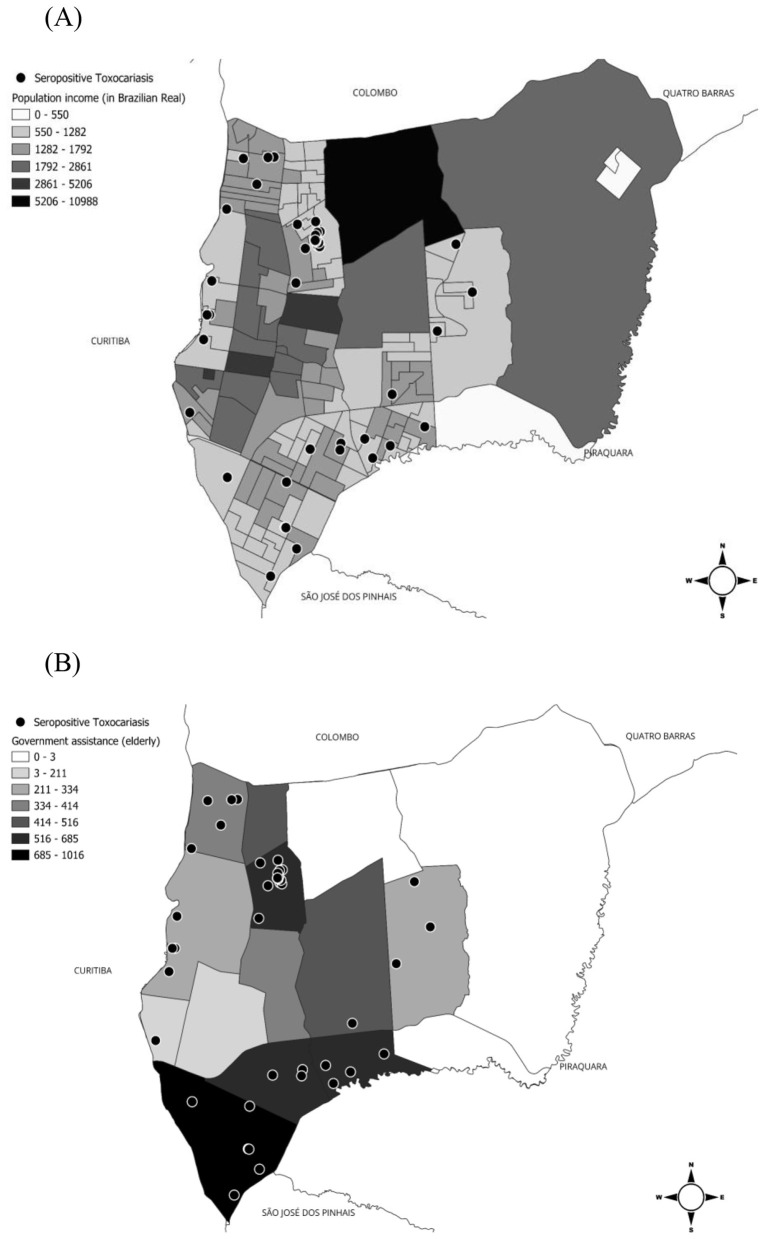
Areas of socioeconomic risk for *Toxocara* spp. in the municipality of Pinhais, southern Brazil. In **(A)**, population income (value of income in Brazilian Real) and in **(B)**, the total number of elderly people receiving government assistance.

Serological results spatially overlapped with family income and the number of elderly people receiving government assistance. A total of 58 of 132 (43.9%) individuals living in lower-income neighborhoods and 37 of 95 (38.9%) individuals living in neighborhoods with a higher number of elderly people receiving government assistance were seropositive (Figure 3).

## 4 Discussion

The present study identified an association between *Toxocara* spp. seropositivity and lower income in the municipality of Pinhais, located in the eighth most populated metropolitan region of southern Brazil.

The municipality of Pinhais is located within the metropolitan area of Curitiba, southern Brazil, which is consideredone of the most sustainable cities in Latin America. In Brazil, Curitiba ranks sixth nationwide in the gross domestic product (GDP) and tenth in the Human Development Index (HDI) (32). Thus, the high HDI (0.751) of Pinhais may not reflect the inherent social vulnerability (22). On the other hand, the social vulnerability index (SVI) measures social determinants of health and more clearly represents the life conditions of such communities (33). In the case of Pinhais, the SVI was considered low (0.261), similar to that of the capital Curitiba (0.253) and within the range of other metropolitan municipalities (average 0.285); however, 1,130 households were considered vulnerable (2.96% of 38,227 households), including those with elderly people, and 0.13% of indivduals lived in households with inadequate water supply and sanitation (33).

The seroprevalence herein (58/132; 43.90%) was higher than that reported among 56 of 280 (20.0%) pregnant women in low-income areas of southeastern Brazil (18). In southern Brazil, the same region as the present study, high seroprevalence rates have been reported among socioeconomically vulnerable groups, including 234 of 370 (63.2%) women inmates (13), 125 of 190 (65.8%) island residents and 87 of 138 (63.0%) seashore mainland residents (15), 247 of 344 (71.8%) adults from a rural population (14), 172 of 208 (82.7%) individuals in *quilombola* communities (12), and 138 of 162 (85.2%) indigenous individuals (11). Recently, the highest seroprevalence (246/258; 95.3%) worldwide was reported in a tri-border indigenous community (Brazil, Paraguay, and Argentina), associated with a large number of infective *Toxocara* spp. eggs found in soil samples from both common (13/42; 30.9%) and household (17/32; 53.1%) areas (8). Although the seroprevalence observed herein was lower than that reported in the aforementioned studies, the present study suggests relatively high population exposure to toxocariasis.

Although age was the only robust individual-level predictor in the adjusted analysis, neighborhoods with the largest proportion of residents earning between half and one and a half Brazilian minimum wages had the highest number of seropositive individuals for *Toxocara* spp. These neighborhoods also had the highest number of elderly people receiving government assistance, highlighting the greater vulnerability of this age group. When the locations of positive samples were compared with the public water supply and sewage collection network, a cluster of positive samples was observed in the Alto Tarumã neighborhood, specifically in an area that had neither system. This finding corroborates previous studies showing that households without connection to the sanitation network are associated with *Toxocara* spp. seropositivity (34). Furthermore, the environmental risk of *Toxocara* spp. contamination may be related to population history and proximity to flooded areas (35). This neighborhood was created to resettle people who previously lived in an occupied area on the banks of the Atuba River and were relocated to this region near the Palmital River. Both areas have a history of flooding, as do other areas in the southern municipality, which also showed positive serological tests. Flooded areas represent a potential risk for the maintenance and spread of human helminthiases, including *Toxocara* spp., depending on the presence of definitive hosts to contaminate the environment (36). Thus, this hypothesis-generating pattern derived from spatial overlays may be useful in future studies with larger sample sizes to better understand associated risk factors.

Indeed, the environment plays a fundamental role in the life cycle of parasites, providing conditions suitable for nutrition, development, and maintenance of infective forms. The influence of temperature and humidity on the development of *Toxocara canis* eggs has been measured under controlled conditions in previous studies, demonstrating the importance of these environmental factors for egg viability (37, 38). For example, laboratory observations showed that 92.9% of eggs kept in a dry environment did not develop larvae, whereas 56.6% of those stored in a humid chamber developed favorably (39). In an analysis of the frequency, density, and environmental factors in soil contamination in public squares in the interior of São Paulo State, Brazil, the variable “rainfall” showed a statistically significant association with the presence of *Toxocara* spp. eggs, reinforcing the hypothesis that humidity favors their maintenance in the soil. Additionally, relative humidity also significantly influenced the total egg count, possibly due to rainfall and consequent soil water retention. Furthermore, a greater number of eggs was observed in areas covered by grasses, which may be related to protection from direct solar radiation and greater moisture retention capacity (40).

In this scenario, riverbank sediments may provide favorable environmental conditions for the survival and spread of *Toxocara* spp. Recent investigations conducted on river sediments in the Brazilian state of Rio Grande do Sul identified the presence of parasites in 65% and 67.5% of the analyzed samples, enompassing 10 species, including *Toxocara canis*, primarily in areas of greater urbanization. This environmental matrix, due to its physical and chemical characteristics, such as pH, humidity, temperature, and nutrient availability, acts as a natural reservoir for pathogens, capable of retaining sanitary waste and effluents, in addition to sustaining infectious and non-infectious forms of parasites until they encounter suitable hosts (41). The banks of artificial water reservoirs have also proven to be environments with potential risk of contamination for animals, human populations, and surrounding ecosystems that rely on these waters. The highest seroprevalence of human toxocariasis in the world (95.3%) was recorded in an indigenous village located on the banks of the Itaipu hydroelectric dam reservoir, near the triple border between Brazil, Paraguay, and Argentina (8). Contaminated soil samples were found at this location, both in common areas (30.9%) and in residential areas (53.1%). The natural dynamics of riverbanks and/or reservoirs are frequently influenced by flooding and environmental variations, such as fluctuations in humidity and temperature, which increase the retention and proliferation of parasitic organisms. The lack of adequate sanitation, combined with densely populated areas living in precarious socioeconomic conditions, exacerbates this situation, as eggs, cysts, and larvae can be ingested by the population through water consumption or contact with contaminated soil.

In this study, variables associated with seropositivity were assessed, and only age was significantly associated with an ELISA reagent test. Logistic regression analysis revealed that elderly individuals (≥53 years) were at higher risk of being seropositive compared to the youngest group (3–20 years). When spatially analyzing the location of seropositive and seronegative individuals, both seropositive individuals at the same address were reported, but these were the older residents who were seropositive. Although children have been considered more susceptible to becoming infected by lower personal hygiene scores or frequent contact with infective *Toxocara* spp. eggs either in contaminated soils (42) or in the hair of dogs or cats (43), some studies have shown a higher frequency of seropositivity in the elderly. A previous study by our research group has highlighted the elderly population as an underreported and neglected group to *Toxocara* spp. infection (17). This study also recommended that the elderly be frequently monitored and that their pets be regularly dewormed to prevent transmission (17). For instance, a study based on the “National Health and Nutrition Examination” database in the United States showed an increase in seroprevalence in people aged over 50 (OR: 2.4), 70 (OR: 2.3), and older than 80 years (OR: 2.6) (17). In southern Brazil, seropositivity was mostly observed in people older than 60 years in a rural population (14), while in *quilombolas*, people over 50 years old had 7.7 times higher odds of seropositivity compared with children (12). High seropositivity in the elderly has been postulated to result from reactivation of *Toxocara* spp. larvae due to immunosenescence and/or the frequent use of medications and immunobiologicals that may lead to an immunocompromised status (14). Another hypothesis is continuous exposure to soil contaminated with *Toxocara* spp. infective eggs (12). In this study, contact with soil was not associated with seropositivity. However, this finding should be interpreted with caution, as recovery of *Toxocara* spp. eggs in household backyards and public recreational areas was not assessed. Additionally, no attempt was made to individually evaluate the health conditions of the participants. On the other hand, although no significant association was observed in this study, onsistent with other studies (44, 45), the majority of individuals reported washing their hands before eating, a personal hygiene practice considered important for reducing the risk of human infection by *Toxocara* spp. (46).

The seropositivity observed in this study was not influenced by gender, in agreement with other serosurveys (15, 47). Nevertheless, being a male has been considered a potential risk factor associated with seropositivity to *Toxocara* spp. according to a global meta-analysis with the general population (48) and children (42). Contact with soil while playing or working has been postulated to justify the highest exposure to infection by children and adults, respectively (7). As already mentioned, soil contact was not associated with seropositivity; however, no attempt was made to investigate the occupation of the adults or the children's recreational behavior.

Other variables related to the transmission of *Toxocara* spp., including contact with dogs, cats, or both, and the habit of consuming raw meat, were not associated with a reagent ELISA test in the present study. A meta-analysis revealed that contact with dogs or cats increases the risk for toxocariasis in younger people (under 18 years) rather than adults (43). However, cases of pleural effusion (49, 50), hepatopathy (50), and hyper-eosinophilic syndrome with multi-organ involvement secondary to persons older than 45 years have been reported in different countries (51). Further, a literature review pointed out that neurotoxocariasis affects predominantly immunocompetent middle-aged males (52). Taking into consideration that the relationships with companion animals have been shown to promote health and wellbeing for older adults (53, 54), and that dogs or cats, particularly dogs kept indoors, are present in the majority of households, preventive measures should be considered to reduce the risks of transmission through direct contact with companion animals. Such measures include prophylactic anthelmintic treatment and proper care regarding the hygiene of animals, particularly puppies (20, 55).

No association with seropositivity was observed in this study regarding the food-borne *Toxocara* spp. transmission. Transmission of *Toxocara* spp. eggs from vegetables and encysted larvae by ingestion of raw meat is considered rare (46), with 27 reported food-borne human toxocariasis cases in the literature (16). To the authors' knowledge, no studies carried out in Brazil have revealed ingestion of raw meat or vegetables as an associated factor for *Toxocara* spp. seropositivity. However, in this study, one-third of the individuals who reported consuming raw meat had a positive ELISA reagent test. Furthermore, vegetables were mostly washed with water. Thus, these factors should be considered when creating educational and preventive measures for toxocariasis for the population.

The selection of households sampled in this study was based on *Rhipicephalus sanguineus* infestation reports. Although *R. sanguineus* was not related to the transmission of *Toxocara* spp., this tick exposure was previously highly associated with socioeconomic factors, particularly in low-income areas with high urban density (21). Thus, a non-related algorithm of vulnerability was initially used to randomly select households, which were visited in order of complaint reports. Nonetheless, this criterion may have directly or indirectly impacted the results, introducing a selection bias that may have limited the representativeness of the study population.

Socioeconomic and environmental indicators were analyzed exclusively at the neighborhood level and were applied only to support contextual and ecological interpretations. These variables were not included in individual-level inferential models, and no individual-level causal inference was attempted, as a result, minimizing the risk of ecological fallacy. In addition, spatial analyses were exploratory in nature and based on aggregated neighborhood-level socioeconomic and environmental data. Thus, the spatial patterns identified should be interpreted as contextual and hypothesis-generating rather than as evidence of causal relationships at the individual level.

Although environmental contamination (e.g., soil sampling) and animal infection/exposure were not directly assessed in this study, the contact with soil and animals was evaluated through an epidemiological questionnaire in a One Health approach.

The present study has limitations, particularly the sample size, which may impair a more robust analysis. Regarding diagnosis, the ELISA test using native TES for detecting IgG antibodies did not allow differentiation between recent and chronic exposure to *Toxocara* spp., limiting the interpretation of the significant seroprevalence observed in the elderly group (21). In this study, no investigation was conducted for the presence of *Toxocara* spp. eggs in soil samples from public parks, schoolchildren's playgrounds, or household areas.

## 5 Conclusion

The present study reported *Toxocara* spp. seropositivity in blood samples from asymptomatic individuals living in socioeconomic vulnerability in the eighth most populated metropolitan region of Brazil, southern Brazil.

Individuals over 53 years of age were at higher risk of seropositivity. The predominance of elderly individuals as a predictor of human toxocariasis revealed a scenario in which cumulative and structural factors outweigh immediate behavioral ones. Therefore, toxocariasis prevention and control strategies should be expanded beyond the traditional focus on children to include adults and the elderly, especially in areas at socio-environmental risk, such as low-income communities with limited access to basic sanitation and a history of flooding.

## Data Availability

The raw data supporting the conclusions of this article will be made available by the authors, without undue reservation.
