# Supplementary material for: Socioeconomic vulnerability associated with toxocariasis exposure in southern Brazil: a One Health approach
Source: Front Public Health. 2026 Mar 18;14:1759431. doi: 10.3389/fpubh.2026.1759431 (PMC13041558; doi:10.3389/fpubh.2026.1759431)

Supplementary Material

Supplementary table 1. Selected variables included in the study

Variable	Level of analysis	Data source	Measurement scale
Seropositivity	Individual	Serological analysis	Categorical (Positive, negative)
Gender	Individual	epidemiological questionnaire	Categorical (Female, male)
Age	Individual	epidemiological questionnaire	Categorical (Age levels)
Dog owner	Individual	epidemiological questionnaire	Categorical (Yes, no)
Cat owner	Individual	epidemiological questionnaire	Categorical (Yes, no)
Dog and cat owner	Individual	epidemiological questionnaire	Categorical (Yes, no)
Dogs living characteristic	Individual	epidemiological questionnaire	Categorical (No dog, stray,

			domiciled)
Washing vegetables before meal	Individual	epidemiological questionnaire	Categorical (Water, sodium hypochlorite, detergent, vinegar)
Washing hands before meals	Individual	epidemiological questionnaire	Categorical (No washing, water, water and soap, soap and alcohol)
Raw meat consumption	Individual	epidemiological questionnaire	Categorical (yes, no)
Contact with soil	Individual	epidemiological questionnaire	Categorical (yes, no)
Leisure and sports areas	neighborhood	municipal database	municipal
flood history	neighborhood	municipal database	municipal
hydrography	neighborhood	municipal database	municipal

sewage networks	neighborhood	municipal database	municipal
forested areas	neighborhood	municipal database	municipal
Permanent Preservation Areas (APP)	neighborhood	municipal database	municipal
Neighborhood boundaries	neighborhood	municipal database	municipal
Municipal borders	neighborhood	municipal database	municipal
Population income	census tracts	IBGE (Censo Demográfico de 2010)	census tracts
Government assistance	neighborhood	municipal database	neighborhood

Supplementary Figure 1. Flowchart of the participant selection and sample collection process.

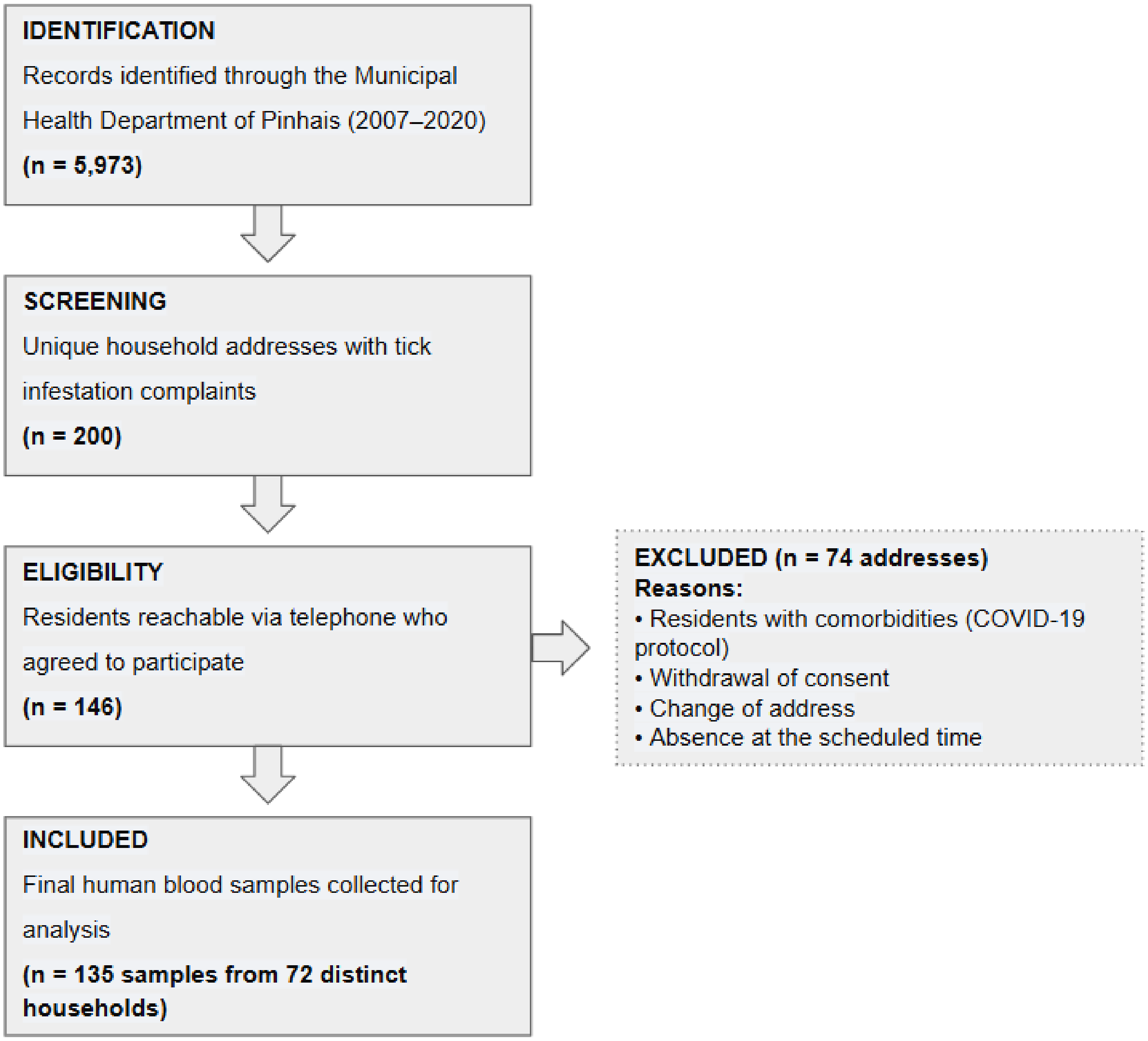

Supplement: Supplementary file 1 [file Data_Sheet_1.pdf]
